# Process‐Based Design of Light Kombucha From Mulberry Coproducts: Effects of Agavins Degree of Polymerization on Physicochemical, Technofunctional, and Functional Potential

**DOI:** 10.1155/ijfo/2076539

**Published:** 2026-04-20

**Authors:** Joshua David Castro-Sánchez, Rosa Isela Ortiz-Basurto, Nuria Elizabeth Rocha-Guzmán, Martha Rocío Moreno-Jiménez, Silvia Marina González-Herrera, Karen Marlenne Herrera-Rocha, Saúl Alberto Álvarez, Blanca Morales-Contreras

**Affiliations:** ^1^ CONAHCYT National Laboratory for Support in the Evaluation of Biotic Products (LaNAEPBi), National Technological Institute of Mexico, Technological Institute of Durango, Blvd. Felipe Pescador 1830 E, Durango, 34080, Mexico; ^2^ Comprehensive Food Research Laboratory, National Technological Institute of Mexico, Technological Institute of Tepic, Tepic, 63175, Nayarit, Mexico

**Keywords:** agavins degree of polymerization, kombucha reformulation, process-based functional design

## Abstract

Reducing residual sugars in kombucha while preserving fermentation performance and consumer acceptance remains a key technological challenge. This study evaluated the effect of partially replacing sucrose with agavins of different degrees of polymerization (DPs) on the physicochemical, technofunctional, and functional properties of kombucha formulated from mulberry coproducts. Formulations were prepared using low DP (LDP), medium DP (MDP), and high DP (HDP) agavins at different substitution levels under controlled fermentation conditions. Beverages were characterized in terms of physicochemical parameters, apparent viscosity, antioxidant capacity, short‐chain fatty acids, and enzyme inhibition. Phenolic profiles were analyzed by LC–MS/MS and interpreted using multivariate approaches (PLS‐DA and PCA). Partial sucrose replacement with LDP agavins at 5% (LDP5%) resulted in a balanced formulation combining enhanced apparent viscosity (0.324 ± 0.008 MPa·s), stable antioxidant capacity across assays, and high sensory acceptability. Microbial populations remained within typical ranges for kombucha fermentation (10^7^–10^8^ CFU/mL), indicating that agavins substitution did not compromise consortium viability. Multivariate analysis suggested that LDP5% was associated with coordinated phenolic remodeling rather than accumulation of terminal fermentation products. These findings indicate that moderate substitution with LDP agavins represents a viable strategy for designing reduced‐sugar kombucha while maintaining technological functionality and sensory quality. Further work is required to directly quantify exopolysaccharide production and assess scalability.

## 1. Introduction

In Mexico, fiscal and front‐of‐pack labeling policies aimed at reducing the consumption of sugar‐sweetened beverages have been associated with measurable reductions in purchasing behavior and purchase intention [1–3]. However, the use of fiscal measures alone may not be sufficient to ensure sustained reductions in sugar intake or obesity prevalence. Recent evidence suggests that while taxes on sugar‐sweetened beverages can reduce purchases, consumers may partially compensate by increasing the consumption of other sugar‐rich substitutes, potentially offsetting the intended health benefits [4, 5]. This highlights the need for complementary strategies, including product reformulation approaches aimed at reducing residual sugars while preserving technological performance and consumer acceptance [6–8].

Within this evolving regulatory and market landscape, kombucha has emerged as a rapidly expanding category of fermented beverages, frequently perceived by consumers as a healthier alternative to conventional soft drinks [9]. Kombucha is produced through the fermentation of a sweetened infusion by a symbiotic culture of bacteria and yeast (SCOBY), resulting in an acidic beverage matrix enriched with organic acids, phenolic derivatives, and other fermentation‐derived metabolites. Despite this growth, kombucha research still faces limited process standardization and strong batch‐to‐batch variability inherent to complex microbial consortia. These limitations have been highlighted in recent studies mapping research trends and methodological gaps in kombucha production [10]. Reported values vary considerably depending on formulation and fermentation conditions. For example, Selvaraj and Gurumurthy [11] estimated an energy value of approximately 15 kcal/100 mL after 12 days of fermentation using an initial sugar concentration of 75 g/L, whereas Luvison et al. [12] reported values ranging from 19.38 to 28.01 kcal/100 mL with 50 g/L sucrose. In contrast, atypically high caloric values, such as those reported for *Clitoria ternatea* kombucha (up to 117 kcal/100 mL), appear to reflect formulation‐specific anomalies rather than representative trends [13]. Notably, most studies prioritize monitoring sugar consumption kinetics rather than directly assessing the caloric implications of the final product, which represents a significant knowledge gap, given the growing demand for kombucha marketed under “low sugar” or “light” claims. In this study, the term “light kombucha” is used operationally to describe formulations with reduced residual sugar content relative to conventional sucrose‐based kombucha beverages, without implying a formal regulatory classification of reduced‐calorie products.

Recent developments in kombucha research can be broadly grouped into two complementary formulation strategies. First, the substitution of *Camellia sinensis* with alternative botanical substrates, including fruits, herbal infusions, and agroindustrial coproducts, has been shown to induce changes in chemical composition and microbial dynamics while maintaining consumer acceptability. Kombucha formulations incorporating pineapple peels, fruit matrices, or mixed botanical substrates have demonstrated consistent modifications in pH, titratable acidity, soluble solids, and bioactive compound profiles [14, 15]. Moreover, synergistic botanical combinations, such as rosemary–guava blends, have been associated with additional functional modulation of the fermented matrix [16]. More recently, studies have expanded this substrate‐diversification approach to nontraditional leaf matrices, supporting the feasibility of alternative foliage substrates for kombucha‐type fermentations and reinforcing sustainability‐oriented sourcing strategies [17, 18]. Within this context, mulberry (*Morus* spp.) leaves and their coproducts have gained attention as promising substrates due to their phenolic richness and technological suitability for fermentation [19–22]. Studies have further reported favorable shifts in SCOBY composition, including increased populations of acetic acid bacteria (AAB) and lactic acid bacteria (LAB) and the recurrent presence of *Komagataeibacter xylinus*, supporting cellulose formation and fermentation stability [19, 21, 23].

A second and increasingly relevant strategy involves the partial or total replacement of sucrose with alternative sweeteners or prebiotic substrates, such as yacon, stevia, monk fruit, or fructan‐rich matrices. These approaches have consistently demonstrated reductions in residual sugars alongside increases in the phenolic content and antioxidant capacity, without compromising sensory acceptance [24–26]. Comparable trends have been reported for fructan‐containing substrates such as *Jerusalem artichoke*, which exhibit sugar utilization patterns and microbial biomass dynamics consistent with prebiotic modulation [27].

Among these alternatives, agavins—branched fructans derived from *Agave* spp.—represent a particularly relevant formulation and process variable. Their fermentability is strongly influenced by the degree of polymerization (DP), which governs microbial accessibility, sugar release kinetics, and metabolic routing during fermentation. Variations in agavins DP have the potential to modulate residual sugar levels, SCOBY activity, colloidal microstructure, and the generation of fermentation‐derived metabolites, including short‐chain fatty acids (SCFAs) and biotransformed phenolic compounds. In the present study, the term *metabiotic* is used in a process‐oriented sense to describe metabolites generated or modified through SCOBY‐mediated fermentation, without implying direct physiological effects *in vivo*.

Accordingly, the objective of this study was to systematically evaluate how partial or total replacement of sucrose with agavins of different DPs (low, medium, and high) influences physicochemical properties, technofunctional behavior, fermentation dynamics, and metabolite profiles in kombucha formulated with mulberry coproducts. A multivariate approach was used to integrate chemical and functional responses and identify optimal formulations. This approach aims to establish reproducible, process‐based criteria for designing “light” kombucha formulations.

## 2. Materials and Methods

### 2.1. Reagents and Agavins Fractions

Unless otherwise stated, reagents were of analytical grade, and chromatographic standards exhibited a purity of ≥ 98%. Solvents used for UPLC–MS/MS analyses were of MS grade, and ultrapure water was obtained using a Milli‐Q purification system. Three agavins fractions differing in DP were employed as formulation variables. The high DP (HDP) fraction was obtained from a standardized commercial source (Biocosechas S.A. de C.V., Mexico) and further fractionated at the Sustainable Agroindustrial Innovation Laboratory (TecNM–Instituto Tecnológico de Tepic) by tangential flow filtration (TFF) using 10 kDa membranes, followed by spray drying. Medium DP (MDP) and low DP (LDP) fractions were acquired as standardized commercial products from NutriAgaves de México S.A. de C.V. and used without further modification. According to supplier specifications, the LDP fraction contained fructooligosaccharides with a DP < 10, the MDP fraction comprised agavins with DP ranging from 10 to 25, and the HDP fraction consisted predominantly of polymers with DP > 30. All fractions contained > 90% total fructans (dry basis), with minor amounts of free sugars (< 9% fructose; < 6% glucose and sucrose combined). These compositional characteristics were provided by the manufacturers and used as the operational basis for DP classification in this study.

### 2.2. Plant Material and SCOBY Activation (First Fermentation, F1)

Mulberry (*Morus* spp.) coproducts, including leaves, petioles, and secondary stems, were collected from greenhouses located in Jacona de Plancarte, Michoacán, Mexico. Sample preparation followed the protocol described by Sariñana‐Núñez et al. [20], including sanitization with 1% (v/v) sodium hypochlorite for 5 min, double rinsing with potable water, oven‐drying at 50°C until moisture content < 12%, grinding to a particle size of 2 mm, and vacuum storage until use.

The kombucha consortium (liquid phase and biofilm) was obtained from a commercial source (Soul Organic Life, Chihuahua, Mexico) and stored under refrigeration. Before experimentation, the SCOBY was activated in a black tea infusion (1.0% w/v) supplemented with sucrose, using 10% (v/v) fermented liquid and 2.5% (w/v) biofilm, incubated at 25°C for 7 days. The F1 was conducted following Rocha‐Guzmán et al. [28] to standardize microbial load; weekly rankings under identical conditions were performed to ensure inoculum consistency.

### 2.3. Second Fermentation (F2)

The F2 was carried out using an infusion of mulberry coproducts (0.25% w/v), prepared according to Sariñana‐Núñez et al. [20]. Fermentations were inoculated with 10% (v/v) activated liquid starter culture and 2.5% (w/w) SCOBY biofilm. All fermentations were conducted statically at 25°C for 11 days, with sampling performed on day 0 and day 11. The experimental design was reproduced across three independent fermentation batches (blocks) conducted on different days, with all 12 formulations prepared in each batch (biological replicates). Fermentations were performed in identical 1‐L glass jars (19 cm height × 10 cm diameter; 7 cm mouth diameter) with a fixed working volume of 600 mL to standardize vessel geometry, headspace, and oxygen exposure. Jars were covered with sterile gauze and secured with elastic bands to allow aerobic fermentation while preventing external contamination.

Sucrose and agavins fractions (LDP, MDP, and HDP) were combined to obtain 12 treatments (Table 1). Substitution formulations were standardized to 10% (w/v) total carbohydrates, whereas S7.5% and S5% represent reduced‐sucrose controls (7.5% and 5.0%, respectively). All concentrations are expressed on a weight/volume basis.

**TABLE 1:** Experimental design and carbohydrate composition of kombucha formulations prepared with sucrose and agavins of different degrees of polymerization.

Trial	Formulation	Sucrose (%)	LDP (%)	MDP (%)	HDP (%)	Total carbohydrate (%)
1	S10%	10.0	0	0	0	10.0
2	S7.5%	7.5	0	0	0	7.5
3	S5%	5.0	0	0	0	5.0
4	LDP10%	0	10.0	0	0	10.0
5	LDP5%	5.0	5.0	0	0	10.0
6	LDP2.5%	7.5	2.5	0	0	10.0
7	MDP10%	0	0	10.0	0	10.0
8	MDP5%	5.0	0	5.0	0	10.0
9	MDP2.5%	7.5	0	2.5	0	10.0
10	HDP10%	0	0	0	10.0	10.0
11	HDP5%	5.0	0	0	5.0	10.0
12	HDP2.5%	7.5	0	0	2.5	10.0

*Note:* S, sucrose; LDP, agavins with a low degree of polymerization; MDP, agavins with a medium degree of polymerization; HDP, agavins with a high degree of polymerization.

### 2.4. SCOBY Establishment and Biofilm Development

On day 11, the SCOBY biofilm was carefully removed and allowed to drain under standardized conditions to minimize excess surface liquid. Wet biofilm weight was recorded as an indirect indicator of consortium establishment and biofilm structuring capacity. Measurements were performed immediately after removal to reduce dehydration‐related variability. Results were expressed as net production Δ g wet biofilm·L^−1^ = final weight – initial weight.

### 2.5. Physicochemical Characterization

#### 2.5.1. pH, Titratable Acidity, and Soluble Solids

The evolution of pH, titratable acidity, and soluble solids (°Brix) was monitored throughout fermentation using standardized methods (NMX‐F‐317‐S‐1978; AOAC 942.15, AOAC 932.12). These parameters were used as primary indicators of substrate consumption, organic acid production, and fermentation stability.

### 2.6. Apparent Viscosity

The flow behavior of all samples was measured at 25°C using a Discovery Hybrid Rheometer 3 (TA Instruments, USA) with a 40‐mm parallel plate setup (SST ST Sand‐Blast ARG2) and a measurement gap of 1000 μm. Before testing, samples were stabilized at 25°C and subjected to a preshear at 30 rad/s for 30 s. Flow curves for all samples were obtained over a range of increasing shear rates (0.1–600 s^−1^). Shear stress (*τ*) and consistency index (*k*), a measure of the apparent viscosity, were recorded as functions of shear rate. The flow curve data were fitted using the power law model,

(1) τ=k·γ˙n,

where *τ* = shear stress (Pa); *k* = consistency index (Pa·s^n^), a measure of the apparent viscosity; $\overset{˙}{\gamma }$ = shear rate (s^−1^); and *n* = flow behavior index (dimensionless).

### 2.7. Determination of Total Soluble Sugars

Total soluble sugars (TSS) were quantified by the phenol–sulfuric acid method according to Dubois et al. [29]. Briefly, samples were centrifuged, and the supernatant was reacted with phenol (5%, w/v) and concentrated sulfuric acid. After color development, absorbance was measured at 490 nm using a UV–Vis spectrophotometer. Quantification was performed using a glucose calibration curve, and results were expressed as mg of glucose equivalents per mL (mg/mL). All analyses were carried out in triplicate.

### 2.8. Antioxidant Capacity

Antioxidant capacity was evaluated using four complementary assays based on different reaction mechanisms: ferric reducing antioxidant power (FRAP) [30], ABTS radical cation scavenging assay [31], DPPH radical scavenging assay [32], and oxygen radical absorbance capacity (ORAC) [33]. All analyses were performed in triplicate. For each assay, calibration curves were built using Trolox as the reference standard, and antioxidant capacity was initially expressed as μmol of Trolox equivalents per mL of beverage (μmol TE/mL).

To enable direct comparison among formulations and across assays with different dynamic ranges, raw antioxidant values were subsequently normalized relative to the process control (S10%), which was set to 1.0 for each antioxidant method. Normalized values were used exclusively for comparative visualization (radar plot) and multivariate analysis, while absolute values were retained for statistical evaluation. This normalization strategy allows for the integration of multiple antioxidant endpoints without bias introduced by assay‐specific scales.

### 2.9. Enzymatic Inhibition

#### 2.9.1. α‐Amylase Inhibition Assay

The inhibitory activity against α‐amylase was determined following a colorimetric method based on starch hydrolysis, as described by Radan et al. [34], with minor adaptations. Briefly, α‐amylase, sample extracts, and potato starch (1% w/v) were prepared in phosphate buffer (0.1 M, pH 6.9). Reaction mixtures containing enzyme and serially diluted samples were preincubated at 37°C, after which starch solution was added to initiate the reaction. Reducing sugars released were quantified using 3,5‐dinitrosalicylic acid (DNS), and absorbance was measured at 540 nm. Inhibitory activity was calculated relative to a control without sample and expressed as phenolic‐equivalent inhibitory activity (mg/mL), reported as quercetin equivalents (QE) and ellagic acid equivalents (EAE) based on external calibration curves.

#### 2.9.2. Pancreatic Lipase Inhibition Assay

Pancreatic lipase inhibition was evaluated according to the method reported by Azadikhah et al. [35]. Reaction mixtures containing sample extracts, pancreatic lipase (1 U/mL), and Tris–HCl buffer (1 M, pH 8.5) were incubated at 37°C. The enzymatic reaction was initiated by adding p‐nitrophenyl butyrate (p‐NPB, 5 mM), and the release of p‐nitrophenol was monitored spectrophotometrically at 412 nm. Lipase inhibitory activity was calculated relative to a blank without sample and expressed as phenolic‐equivalent inhibitory activity (mg/mL), reported as QE and EAE derived from external calibration curves.

### 2.10. Targeted LC–MS/MS Profiling of Phenolic Metabolites

Phenolic profiling was performed by ultrahigh‐performance liquid chromatography coupled to tandem mass spectrometry (UPLC–MS/MS) using a targeted MRM‐based strategy. The method was adapted from previously reported conditions for phenolic compound analysis in complex matrices [20]. Analyses were conducted on a Waters ACQUITY Class‐H UPLC system coupled to a Xevo TQ‐S triple quadrupole mass spectrometer equipped with an electrospray ionization (ESI) source.

Chromatographic separation was achieved using a reversed‐phase ACQUITY BEH C18 column (1.7 μm particle size, 50 × 2.1 mm i.d.), operated at 35°C and a constant flow rate of 0.250 mL·min^−1^. The autosampler temperature was maintained at 6°C to preserve sample stability throughout the analytical sequence. The mobile phase consisted of a binary solvent system comprising 7.5 mM formic acid in Milli‐Q water (Solvent A) and acetonitrile (Solvent B). Elution was carried out using a stepwise gradient program, increasing Solvent B from 3% to 9%, 16%, and 50% at 0.00, 1.23, 3.82, and 11.40 min, respectively, followed by re‐equilibration at 3% Solvent B until 13.24 min. An additional stabilization period of 2.76 min at initial conditions was applied prior to subsequent injections to ensure chromatographic reproducibility, following the method described by Díaz‐Rivas et al. [36].

Mass spectrometric detection was conducted in the negative ESI mode (ESI^−^), using nitrogen as the nebulizing and desolvation gas. The capillary voltage was set at 2.5 kV, with a collision gas flow of 0.13 mL·min^−1^. Collision energies were optimized and fixed at 5.0 eV for the MS mode and 20.0 eV for MS/MS acquisition. The desolvation and source temperatures were maintained at 400°C and 150°C, respectively.

Data acquisition was performed in the multiple reaction monitoring (MRM) mode to enable selective and sensitive detection of targeted phenolic compounds. Compound identification was based on matching retention times, characteristic fragmentation patterns, and comparison with authentic analytical standards when available. Quantification was performed using external calibration curves, and the results were grouped according to phenolic subclasses (hydroxybenzoic acids, hydroxycinnamic acids, flavonoids, and hydrolyzable tannins) to facilitate comparative and process‐based interpretation across formulations. Compounds lacking analytical standards were tentatively identified based on retention time alignment, MRM transitions, and fragmentation patterns and were semiquantitatively estimated using structurally related reference compounds.

### 2.11. Targeted LC–MS/MS Profiling of (SCFAs)

SCFAs in kombucha samples were analyzed by liquid chromatography coupled to ESI tandem mass spectrometry (LC–ESI–MS/MS) following a chemical derivatization approach with 3‐nitrophenylhydrazine (3‐NPH) [37]. For each analysis, 40 μL of liquid sample was transferred to a reaction vial and mixed with 40 μL of 3‐NPH solution (200 mM) and 40 μL of EDC solution (120 mM). Subsequently, 20 μL of pyridine (6%, v/v) was added to promote derivatization. The reaction mixture was incubated at 30°C for 30 min. After completion of the derivatization reaction, samples were filtered through 0.45 μm membranes and directly subjected to LC–MS/MS analysis.

Chromatographic separation was performed using an ACQUITY UPLC system (Waters Corp.) equipped with a HSS C18 column (2.1 × 100 mm, 1.7 μm particle size), maintained at 40°C. The autosampler temperature was set at 6°C. The injection volume was 0.1 μL, and the total run time was 15 min. The mobile phases consisted of water containing formic acid (100:0.01, v/v; phase A) and acetonitrile containing formic acid (100:0.01, v/v; phase B). Elution was carried out at a constant flow rate of 0.350 mL·min^−1^ using a gradient program as follows: 85% A/15% B from 0 to 2.0 min; maintained at 85% A/15% B until 9.0 min; shifted to 45% A/55% B from 9.0 to 10.0 min; held at 45% A/55% B until 13.0 min; and returned to initial conditions (85% A/15% B) from 13.0 to 15.0 min for column re‐equilibration.

Mass spectrometric detection was performed on a triple quadrupole mass spectrometer operated in ESI^−^. The capillary voltage was set at 4.2 kV, cone voltage at 30 V, and source offset at 60 V. Source and desolvation temperatures were maintained at 150°C and 450°C, respectively. Nitrogen was used as nebulizing and desolvation gas, with flow rates of 7.0 bar and 500 L·h^−1^, respectively, while the cone gas flow was set at 150 L·h^−1^. The collision gas flow was maintained at 0.13 mL·min^−1^. Quadrupole resolutions were set to 2.8 (LM) and 14.9 (HM) for both Q1 and Q3. Collision energies were fixed at 2 eV in the MS mode and 20 eV in the MS/MS mode. Mass acquisition was performed over a range of m/z 100–300, using a scan time of 0.5 s and a collision cell energy ramp from −120 to −10 V.

SCFAs were monitored in the MRM mode using compound‐specific transitions optimized for 3‐NPH derivatives. The monitored compounds included acetate (194.1 > 137.1), propionate (208.1 > 165.1), isobutyrate/butyrate (222.1 > 137.1), 2‐methylbutyrate/isovalerate/valerate (236.1 > 137.1), and 3‐methylvalerate/isocaproate/caproate (250.1 > 137.1).

### 2.12. Determination of Microbial Populations

Microbial populations associated with kombucha fermentation were determined by culture‐dependent methods. The microbial groups evaluated included yeasts, AAB, LAB, and bifidobacteria. For each selected formulation, 10 mL of the fermented beverage was aseptically collected and subjected to serial decimal dilutions using sterile diluent. Appropriate dilutions, ranging from 10^−4^ to 10^−8^, were prepared for microbial enumeration.

Aliquots of the corresponding dilutions were plated on selective culture media according to the target microbial group. De Man, Rogosa, and Sharpe (MRS) agar was used for the enumeration of LAB, while MRS agar supplemented with cysteine was employed for bifidobacteria. AAB were enumerated on a mannitol agar medium, and yeasts were quantified using yeast extract–peptone–dextrose (YPD) agar.

Plates were incubated under conditions specific to each microbial group. LAB and bifidobacteria were incubated at 37 ± 2°C under anaerobic conditions, yeasts were incubated at 30 ± 2°C under aerobic conditions, and AAB were incubated at 25 ± 2°C. After the appropriate incubation period, colonies were counted, and microbial populations were expressed as the logarithm of colony‐forming units per milliliter (log CFU·mL^−1^).

Microbial enumeration was used to contextualize fermentation performance and consortium balance under selected optimal formulations, rather than to infer probiotic viability.

### 2.13. Sensory Analysis

Sensory evaluation was conducted in a controlled sensory analysis room under a single‐blind experimental design. Samples were prepared immediately before evaluation and served at 4°C in portions of 30 mL. To ensure objectivity and minimize bias, each sample was identified using alphanumeric codes and presented to panelists following a randomized serving order. Between tests, panelists followed a palate‐cleansing protocol consisting of rinsing with room‐temperature water and consumption of plain, unsalted crackers. A resting period of 1 minute was imposed between samples to reduce sensory fatigue and carry‐over effects.

The sensory panel consisted of 10 trained panelists. Participants were included if they were regular consumers of fermented beverages and reported no known allergies or conditions affecting taste perception. Individuals reporting illness, taste or smell disorders, or allergies to the ingredients used in the samples were excluded from the evaluation. Before formal testing, panelists completed orientation and calibration sessions focused on attribute recognition, scale familiarization, and consensus‐based refinement of descriptors and their operational definitions. Panel reproducibility was supported by standardized evaluation conditions, randomized sample presentation, and consensus agreement on attribute definitions to minimize interpanel variability. The evaluation followed a three‐stage protocol. In the first stage, free choice profiling (FCP) was applied to capture the initial sensory descriptors perceived in the samples. Panelists freely generated terms describing appearance, aroma, flavor, and mouthfeel, allowing an unbiased identification of the most relevant sensory attributes.

In the second stage, a quantitative descriptive analysis (QDA) was performed. The same 10 trained panelists evaluated the intensity of the previously defined attributes using a structured nine‐point intensity scale, where 1 corresponded to “Not Perceptible” and 9 to “Extremely Intense.” This stage provided a quantitative description of the sensory profile of the selected formulations.

In the third stage, directed group evaluation was carried out to assess product acceptability. Overall liking was rated using a nine‐point hedonic scale ranging from 1 (“Dislike Extremely”) to 9 (“Like Extremely”). This phase aimed to identify acceptance trends among formulations previously identified as optimal based on physicochemical, technofunctional, and antioxidant criteria.

### 2.14. Statistical Analysis

All experiments were performed in triplicate, and the results are expressed as mean ± SD. Fermentations were conducted in three independent batches on different days and treated as biological replicates. The potential batch effect was evaluated by including “batch” as a factor in a two‐way ANOVA (treatment and batch); as neither the batch effect nor the treatment × batch interaction was statistically significant (*p* > 0.05), data were pooled across batches for subsequent analyses. Univariate comparisons among formulations were conducted by one‐way ANOVA, followed by Dunnett’s post hoc test using the process control (S10%) as reference (*p* < 0.05), as the formulation matrix (Table 1) represents a structured but nonorthogonal multifactor design, and primary inference was based on planned control‐referenced contrasts. Antioxidant assay results were normalized to S10% (set to 1.0) for comparative visualization. Multivariate analyses included PCA for integrative pattern recognition and partial least squares discriminant analysis (PLS‐DA) of phenolic profiles, using autoscaled data; discriminant variables were selected based on VIP ≥ 1.0. Model performance was assessed by *R* ^2^ and *Q* ^2^, with *Q* ^2^ derived from fivefold cross‐validation implemented in MetaboAnalyst. Model robustness was further evaluated by permutation testing (1000 permutations), and empirical *p* values were calculated to assess the likelihood of class separation under random label assignment. Group differences were validated by PERMANOVA (999 permutations). All experimental measurements were obtained for each treatment and replicate, and no missing data were observed; therefore, no data imputation procedures were required. Analyses were conducted using IBM SPSS Statistics 22 (IBM Corp., Armonk, NY, USA) and MetaboAnalyst 6.0.

## 3. Results and Discussion

**FIGURE 1:**
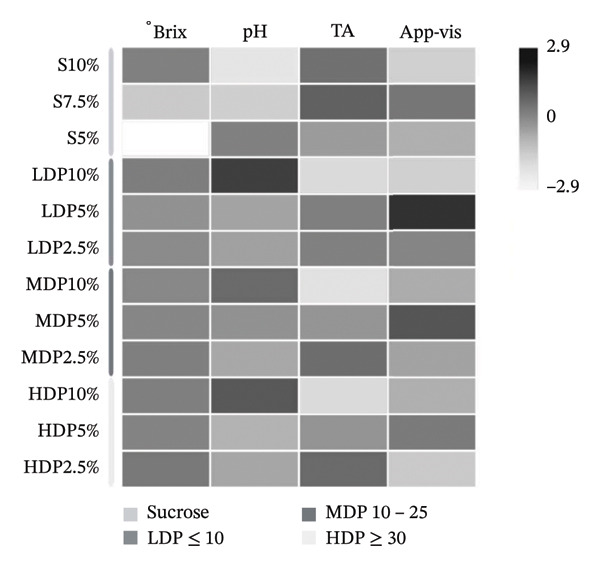
Heatmap illustrates the standardized variation (z‐scores) of physicochemical parameters (°Brix, pH, and titratable acidity) and apparent viscosity in kombucha analogs formulated with different sucrose levels and agavins with distinct degrees of polymerization (LDP, MDP, and HDP). Color intensity reflects relative increases or decreases across formulations. °Brix, total soluble solids; TA, titratable acidity; App‐Vis, apparent viscosity; S, sucrose; LDP, agavins with a low degree of polymerization; MDP, agavins with a medium degree of polymerization; and HDP, agavins with a high degree of polymerization.

The physicochemical and technofunctional characterization revealed distinct responses depending on both the type and proportion of sweetener used (Figure 1). Total soluble solids (°Brix) did not show pronounced divergence among formulations. This suggests broadly comparable levels of soluble solids across the evaluation time points. However, °Brix is an indirect proxy based on the refractive index and may be influenced by solute composition (e.g., sucrose versus fructan‐rich agavins fractions). Therefore, its apparent stability should be interpreted cautiously and does not necessarily indicate equivalent carbohydrate depletion across treatments. These observations suggest that partial sucrose replacement did not markedly alter bulk soluble solids, but rather modulated downstream fermentative and technofunctional responses.

In contrast, pH and titratable acidity were more sensitive to the substitution strategy. Formulations with partial sucrose replacement exhibited lower final pH values, reflecting enhanced acidification. The coexistence of sucrose as a readily fermentable carbon source with agavins likely sustained the metabolic activity of AAB and yeasts. This interaction may have promoted organic acid production without compromising fermentation performance, as reported for mixed‐carbohydrate systems.

Apparent viscosity emerged as the most discriminant technofunctional parameter. Formulations containing 5% sucrose combined with 5% LDP or MDP agavins showed higher viscosity compared with sucrose‐only controls. This response indicates a more structured colloidal matrix, consistent with the combined contribution of insoluble bacterial cellulose fragments and soluble exopolysaccharides produced by the kombucha consortium. SCOBY systems are known to generate both structural cellulose and soluble EPS, mainly fructan‐type polymers, which jointly contribute to beverage body and mouthfeel [38, 39].

To support the rheological interpretation, flow curves were fitted to the power law model, and the consistency index (*k*) and flow behavior index (*n*) are reported (Table 2). All formulations exhibited *n* < 1, confirming pseudoplastic (shear‐thinning) behavior rather than Newtonian flow, with high goodness of fit (*R* ^2^).

**TABLE 2:** Power law model parameters describing the flow behavior of kombucha analogs formulated with sucrose and agavins of different degrees of polymerization.

Treatment	*k* (Pa·s^n^)	*n*	*R* ^2^
S10%	0.265 ± 0.006^a^	0.738 ± 0.006^a^	0.985 ± 0.0005^a^
S7.5%	0.313 ± 0.002^ab^	0.754 ± 0.004^a^	0.991 ± 0.0001^bc^
S5%	0.323 ± 0.027^ab^	0.767 ± 0.009^a^	0.992 ± 0.0010^c^
LDP 10%	0.285 ± 0.045^ab^	0.751 ± 0.032^a^	0.986 ± 0.0040^ab^
LDP5%	0.324 ± 0.008^b^	0.751 ± 0.006^a^	0.992 ± 0.0010^c^
LDP2.5%	0.300 ± 0.010^ab^	0.747 ± 0.004^a^	0.991 ± 0.0010^bc^
MDP10%	0.281 ± 0.024^ab^	0.743 ± 0.03^a^	0.988 ± 0.0005^abc^
MDP5%	0.309 ± 0.013^ab^	0.744 ± 0.004^a^	0.991 ± 0.0010^c^
MDP2.5%	0.298 ± 0.002^ab^	0.753 ± 0.007^a^	0.989 ± 0.0005^abc^
HDP10%	0.266 ± 0.015^ab^	0.729 ± 0.009^a^	0.986 ± 0.0005^ab^
HDP5%	0.296 ± 0.026^ab^	0.744 ± 0.009^a^	0.989 ± 0.0020^abc^
HDP2.5%	0.287 ± 0.003^ab^	0.746 ± 0.005^a^	0.989 ± 0.0010^abc^

*Note:* Values are expressed as mean ± standard deviation (*n* = 3). Different superscript letters within the same column indicate significant differences among treatments according to one‐way ANOVA followed by Tukey’s HSD test (*p* < 0.05). *k*, consistency coefficient; *n*, flow behavior index; *R* ^2^, coefficient of determination; S, sucrose; LDP, agavins with a low degree of polymerization; MDP, agavins with a medium degree of polymerization; HDP, agavins with a high degree of polymerization.

Net SCOBY production (Δ SCOBY) exhibited treatment‐dependent variability (Figure 2(a)). The process control (S10%) showed intermediate biofilm formation (33.33 ± 3.21), comparable to most formulations containing MDP and HDP agavins as well as partial substitutions at 7.5%. The overall one‐way ANOVA did not detect statistically significant differences among treatments (*p* > 0.05). However, because the primary analytical interest involved comparison of each formulation against the process control (S10%), Dunnett’s post hoc test was performed as a planned contrast. Under this framework, the LDP5% formulation exhibited a significantly lower Δ SCOBY relative to S10% (Dunnett’s, *p* < 0.05). This result should therefore be interpreted as a control‐focused comparison rather than evidence of a global treatment effect across all formulations.

FIGURE 2Net SCOBY production (Δ SCOBY) (a) and total soluble sugars (TSS) (b) in kombucha analogs formulated with sucrose and agavins of different degrees of polymerization. Statistical comparisons were performed using Dunnett’s test with the process control (S10%) as reference. *p* < 0.05 (^∗^), *p* < 0.01 (^∗∗^), and *p* < 0.001 (^∗∗∗^) indicate significant differences relative to the control (S10%). S, sucrose; LDP, agavins with a low degree of polymerization; MDP, agavins with a medium degree of polymerization; HDP, agavins with a high degree of polymerization.

**Figure:**
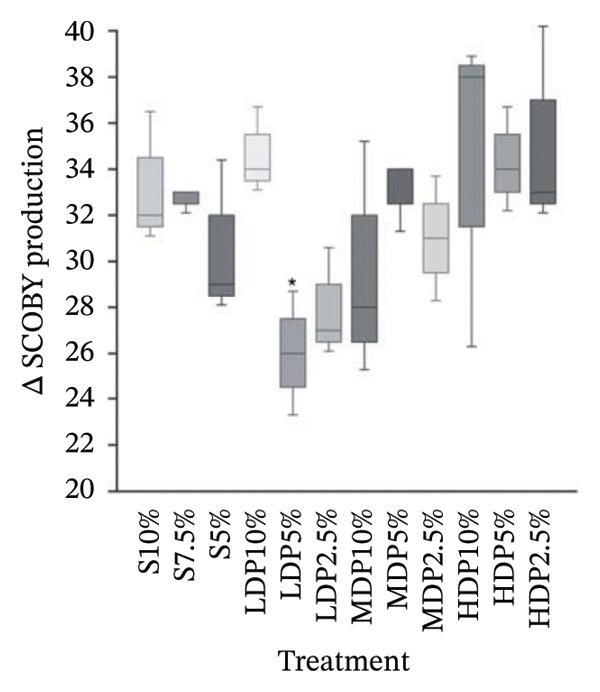
(a)

**Figure:**
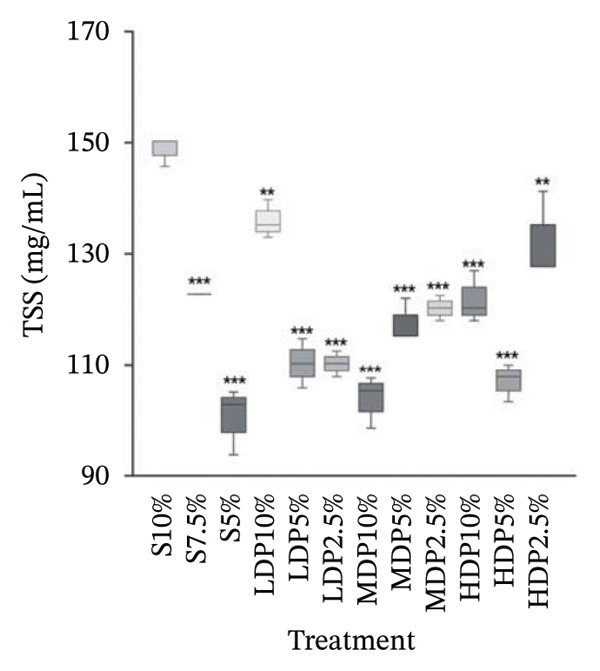
(b)

Notably, the LDP5% formulation combined the lowest Δ SCOBY values with the highest apparent viscosity. When Δ SCOBY was analyzed together with TSS (Figure 2(b)), this response pattern supports a functional decoupling between biofilm formation (insoluble bacterial cellulose) and liquid‐phase structuring. Under partial sucrose replacement with LDP agavins, carbon flux within the kombucha consortium appears to be preferentially redirected toward soluble or colloidal components rather than toward pellicle assembly. The significant differences observed in TSS relative to S10% further indicate that carbohydrate availability and transformation are closely linked to this redistribution. This shift appears to favor viscosity‐enhancing soluble exopolysaccharides rather than insoluble SCOBY biomass. This behavior is consistent with previous studies reporting that kombucha fermentation produces fructan‐type exopolysaccharides whose concentration and structure vary depending on the fermentation substrate and microbial composition [39].

The inverse trend between residual soluble sugars and apparent viscosity further supports this interpretation. This pattern suggests that carbohydrate transformation enhances the colloidal structuring of the beverage without proportional increases in SCOBY biomass. A similar behavior was reported in other fermented beverages, where soluble EPS production by AAB and LAB enhances viscosity and sensory attributes while limiting the accumulation of insoluble matrices [40, 41].

Overall, relative to the process control (S10%), partial sucrose replacement at 5% with LDP agavins does not maximize SCOBY production but promotes a favorable rheological and technofunctional profile. This balance supports its selection as a robust technological strategy for developing reduced‐calorie kombucha analogs with improved structural complexity and functional attributes.

**FIGURE 3:**
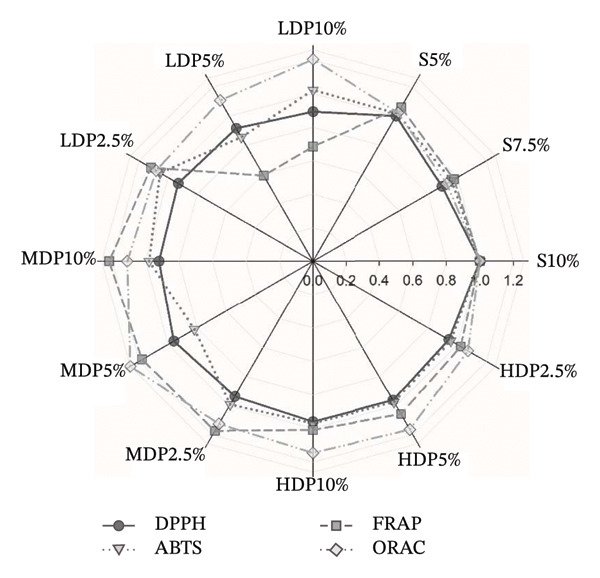
Radar plot of antioxidant capacity of kombucha analogs determined by DPPH, ABTS, FRAP, and ORAC assays. Data were normalized relative to the process control (S10%), which was set to 1.0 for all responses, to allow for comparison among assays with different dynamic ranges. The plot highlights differentiated antioxidant response patterns associated with partial sucrose replacement and agavins degree of polymerization. S, sucrose; LDP, agavins with a low degree of polymerization; MDP, agavins with a medium degree of polymerization; and HDP, agavins with a high degree of polymerization.

The antioxidant capacity of kombucha analogs differed among formulations depending on the type and proportion of sweetener used (Figure 3). Across treatments, ORAC values (peroxyl radical scavenging capacity) exhibited the widest dynamic range. Formulations with partial sucrose replacement using LDP and MDP agavins showed higher ORAC responses compared with sucrose‐only controls. In contrast, electron transfer–based assays (DPPH, ABTS, and FRAP) displayed more moderate variations among treatments, with trends that were assay‐specific rather than uniform.

Formulations incorporating partial sucrose replacement generally maintained or enhanced antioxidant responses relative to the process control, indicating that substitution did not compromise antioxidant potential. Notably, some formulations exhibited divergent behaviors between assays, with higher ORAC values not always accompanied by proportional increases in DPPH, ABTS, or FRAP, highlighting the contribution of different antioxidant mechanisms.

This differentiated antioxidant response is consistent with previous reports, indicating that the type and complexity of the carbohydrate substrate modulate antioxidant outcomes in a kombucha analog in an assay‐dependent manner. ORAC values reflect peroxyl radical scavenging capacity through hydrogen atom transfer mechanisms. This assay is often more sensitive to changes in polyphenolic composition and fermentation‐derived metabolites than electron‐transfer–based assays such as DPPH, ABTS, or FRAP.

Studies evaluating sucrose replacement with alternative sugars or complex substrates in kombucha and related matrices have reported enhanced antioxidant responses, particularly for DPPH and FRAP. These findings support the role of substrate quality beyond the total sugar concentration [42, 43]. Similarly, enrichment with polyphenol‐rich cosubstrates has been shown to amplify antioxidant capacity during kombucha fermentation, reinforcing the contribution of phenolic composition to assay‐specific outcomes [14, 44]. Together, these findings support that partial sucrose replacement modulates antioxidant performance through changes in both phenolic availability and fermentation‐mediated biotransformation, rather than through simple dilution or concentration effects.

Comparable mechanisms have been proposed in other fermented beverages, where synergistic interactions between polyphenols and fermentation‐derived exopolysaccharides enhance radical scavenging capacity through matrix stabilization and improved polyphenol retention. Although these effects are well documented in nontea fermented systems, direct experimental evidence in kombucha remains limited [40].

As shown in Table 3, partial sucrose replacement by agavins resulted in differentiated inhibitory responses against α‐amylase and pancreatic lipase, indicating that enzymatic modulation was not uniformly enhanced across digestive pathways. Because inhibition was evaluated under a fixed assay condition (single test concentration), results are reported as phenolic‐equivalent inhibitory activity (mg/mL). Values are expressed as QE and EAE derived from external calibration curves. Accordingly, these values should be interpreted as standardized “equivalent” outputs rather than IC_50_ values or concentration–response–based percent inhibition. All formulations exhibited significant α‐amylase inhibitory equivalents relative to the sucrose control (S10%). However, LDP5% showed a consistent and moderate inhibitory effect, representing a more balanced profile than the lower activity observed in the control or the stronger inhibition detected at higher substitution levels. In contrast, pancreatic lipase inhibitory equivalents remained comparatively low and only modestly modulated among treatments, suggesting a selective impact on carbohydrate‐hydrolyzing enzymes rather than a generalized suppression of digestive activity. QE and EAE were reported to provide complementary reference scaling for two prevalent phenolic chemotypes in kombucha‐type matrices: flavonoid‐like structures (quercetin) and ellagic acid–type hydrolyzable tannin derivatives. This approach improves interpretability across formulations with distinct phenolic remodeling patterns.

**TABLE 3:** α‐Amylase and pancreatic lipase inhibitory activity of kombucha analogs formulated with sucrose reduction or replacement by agavins of different degrees of polymerization.

Treatment	α‐Amylase inhibition		Pancreatic lipase inhibition	
	Quercetin	Ellagic acid	Quercetin	Ellagic acid
S10%	58.5 ± 2.9	178.4 ± 8.6	2.2 ± 0.1	13.7 ± 0.5
S7.5%	43.0 ± 2.1^∗∗^	132.1 ± 6.3^∗∗^	3.2 ± 0.4^∗∗∗^	18.3 ± 1.9^∗∗∗^
S5%	27.7 ± 1.2^∗∗∗^	86.7 ± 3.5^∗∗∗^	2.6 ± 0.3	15.3 ± 1.4
LDP10%	36.1 ± 1.8^∗∗∗^	111.8 ± 5.5^∗∗∗^	3.4 ± 0.1^∗∗∗^	18.9 ± 0.3^∗∗∗^
LDP5%	40.8 ± 1.4^∗∗∗^	125.6 ± 4.2^∗∗∗^	2.3 ± 0.0	14.3 ± 0.2
LDP2.5%	40.3 ± 2.1^∗∗∗^	124.3 ± 6.2^∗∗∗^	4.0 ± 0.4^∗∗∗^	21.9 ± 1.9^∗∗∗^
MDP10%	33.5 ± 2.3^∗∗^	103.9 ± 7.0^∗∗∗^	1.7 ± 0.1^∗∗^	11.2 ± 0.3^∗∗^
MDP5%	36.0 ± 2.1^∗∗∗^	111.3 ± 6.3^∗∗∗^	2.2 ± 0.0	13.8 ± 0.1
MDP2.5%	29.3 ± 1.1^∗∗∗^	91.6 ± 3.3^∗∗∗^	2.3 ± 0.1	14.0 ± 0.5
HDP10%	14.5 ± 1.1^∗∗∗^	47.5 ± 3.3^∗∗∗^	2.8 ± 0.0	16.6 ± 0.2
HDP5%	35.5 ± 0.8^∗∗∗^	110.0 ± 2.3^∗∗∗^	2.8 ± 0.1	16.4 ± 0.4
HDP2.5%	44.8 ± 1.9^∗∗∗^	137.5 ± 5.5^∗∗∗^	2.9 ± 0.4^∗^	16.8 ± 1.9^∗^

*Note:* Values are expressed as mean ± SD (*n* = 3). Statistical differences were evaluated using one‐way ANOVA followed by Dunnett’s post hoc test, with S10% as the control. *p* < 0.05 (^∗^), *p* < 0.01 (^∗∗^), and *p* < 0.001 (^∗∗∗^) indicate significant differences relative to the control (S10%). Inhibitory activity is expressed as equivalent inhibition based on quercetin or ellagic acid calibration curves. S, sucrose; LDP, agavins with a low degree of polymerization; MDP, agavins with a medium degree of polymerization; and HDP, agavins with a high degree of polymerization.

This behavior is consistent with previous reports showing that phenolic compounds of low to intermediate molecular size, including ellagic acid derivatives and related hydrolyzable tannins, exert moderate α‐amylase inhibition. In contrast, substantially stronger inhibition is typically associated with high‐molecular‐weight ellagitannins, gallotannins, or highly polymerized proanthocyanidins [45–47]. Conversely, pancreatic lipase inhibition has been shown to depend more strongly on large, multidentate polyphenols, such as gallotannins, whereas simpler phenolic acids and LDP derivatives display limited activity [48, 49]. In this context, the comparatively low and weakly modulated lipase‐equivalent values across treatments are consistent with the absence of direct evidence in the present dataset for abundant high‐molecular‐weight multidentate inhibitors.

Therefore, the enzymatic profile observed for LDP5% is consistent with a rebalancing rather than maximization of inhibitory capacity, supporting a formulation that modulates starch digestion without excessively impairing lipid hydrolysis. The specific phenolic contributors underlying this selective inhibition pattern are examined in detail in the subsequent multivariate analysis of phenolic composition.

The differentiated inhibitory patterns observed for α‐amylase and pancreatic lipase are consistent with the technofunctional responses described. Formulations such as LDP5%, which exhibited reduced net SCOBY production (Figure 2(b)) and increased apparent viscosity (Figure 1), also showed attenuated α‐amylase inhibition profile (Table 3) and a comparatively low, only modestly modulated lipase response across treatments. These observations are consistent with the carbon allocation pattern discussed above. However, because EPS were not directly quantified in the present study, this mechanism should be considered a hypothesis that requires targeted EPS characterization (e.g., gravimetric/HPSEC quantification) for confirmation.

It is important to note that the use of phenolic reference standards rather than pharmaceutical inhibitors reinforces the physiological relevance of the results. Accordingly, the inhibitory activities observed reflect mechanisms compatible with food systems rather than pharmacological extremes. This approach is consistent with the functional framework of kombucha‐like foods in terms of polyphenolic content and profile.

To clarify which phenolic constituents drove formulation‐dependent differences, a supervised PLS‐DA was built from the LC–MS phenolic fingerprint (Figure 4). From > 45 detected compounds, 15 features were retained as discriminant markers using VIP ≥ 1.0, consistent with established marker‐selection practices in complex fermented matrices [50–52]. The model showed strong fit and predictive performance (*R* ^2^ = 0.943; *Q* ^2^ = 0.798), with *Q* ^2^ obtained through fivefold cross‐validation. Model robustness was further evaluated by permutation testing (1000 permutations), in which the observed discrimination significantly exceeded that generated under random class assignments (empirical *p* = 0.001), supporting the stability of the supervised classification.

FIGURE 4Partial least squares discriminant analysis (PLS‐DA) of phenolic constituents in kombucha analogs formulated with partial sucrose replacement by agavins of different degrees of polymerization. (a) Variable importance in projection (VIP) scores highlighting the phenolic compounds contributing most to sample discrimination: coumaric acid (CouA), ellagic acid rhamnoside (EAR), rutin (R), shikimic acid (ShA), quercetin sulfoglucuronide (Sglur‐Q), quinic acid (QA), trihydroxybenzaldehyde (THBA), ellagic acid (EA), caffeic acid glucuronide (Ca‐glur), trans‐cinnamic acid (t‐CiA), quercetin glucoside (Q‐glu), quercetin glucuronide (Q‐glur), protocatechuic acid (PA), epicatechin gallate (ECG), and caffeic acid (CaA). (b) PLS‐DA biplot (scores–loadings) illustrating the multivariate separation of formulations according to agavins structure and substitution level. S, sucrose; LDP, agavins with a low degree of polymerization; MDP, agavins with a medium degree of polymerization; and HDP, agavins with a high degree of polymerization.

**Figure:**
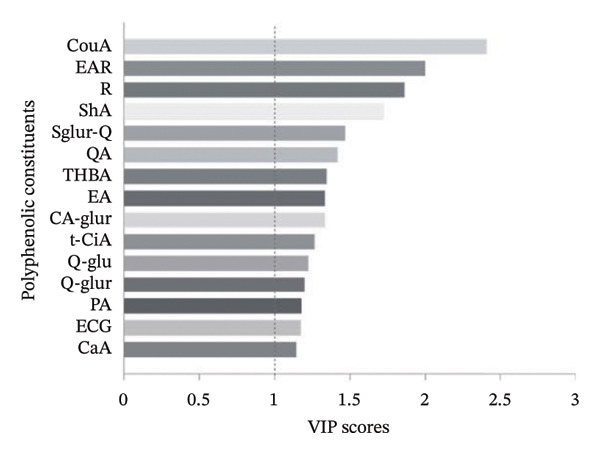
(a)

**Figure:**
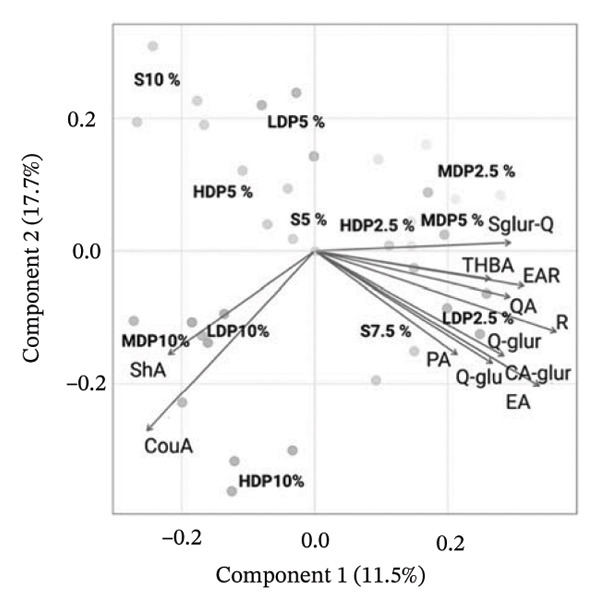
(b)

The biplot indicates that discrimination was driven by specific phenolic classes—flavonoid glycosides, phenolic acids, and hydrolyzable tannin derivatives—rather than by bulk phenolic abundance. Notably, 5% agavins formulations (particularly LDP5% and MDP5%) aligned with a coherent signature dominated by quercetin‐derived glycosides (quercetin sulfoglucuronide, quercetin glucuronide, and quercetin glucoside), ellagic acid derivatives (ellagic acid rhamnoside, ellagic acid methyl ester), and low‐molecular‐weight phenolic acids (chlorogenic acid, quinic acid, trans‐cinnamic acid, protocatechuic acid, and caffeic acid glucuronide). This profile is compatible with reported microbial remodeling routes of tea polyphenols during kombucha fermentation, including deglycosylation, ester hydrolysis, and oxidative rearrangements [53, 54]. In contrast, 2.5% substitution treatments clustered closer to the origin, whereas higher substitution levels and the sucrose control displayed more dispersed loadings. This pattern indicates that partial sucrose replacement at 5% favored coordinated phenolic reorganization rather than uniform accumulation—an effect consistent with PLS‐DA‐based differentiation reported for kombucha and other fermented beverages [50, 51, 55].

**FIGURE 5:**
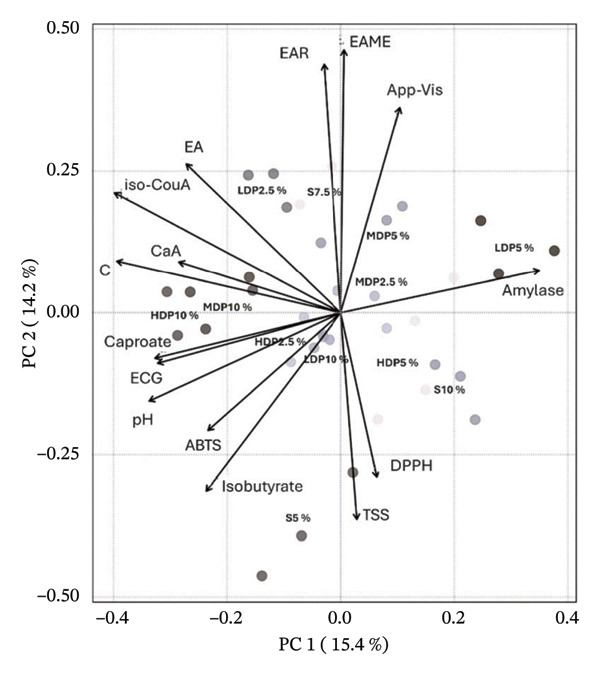
Principal component analysis (PCA) biplot integrating physicochemical parameters, antioxidant capacity, enzymatic activity, short‐chain fatty acids, and the most discriminant phenolic metabolites of kombucha analogs. PCA was performed on autoscaled (mean‐centered and unit variance–scaled) data before model construction. The score plot illustrates sample distribution according to formulation and agavins degree of polymerization, while loading vectors represent the contribution of key variables, including phenolic metabolites identified as discriminant compounds. The first two principal components (PC1 and PC2) explain 15.4% and 14.2% of the total variance, respectively. Multivariate differences among formulations were statistically supported by PERMANOVA (*F* = 18.132, *R* ^2^ = 0.8926, *p* = 0.001; 999 permutations), evidencing the strong effect of partial sucrose replacement and agavins structure on the global metabolic and physicochemical profile. App‐Vis, apparent viscosity; TSS, total soluble sugars; EAR, ellagic acid rhamnoside; EAME, ellagic acid methyl ester; EA, ellagic acid; Iso‐CouA, iso‐coumaric acid; CaA, caffeic acid; C, catechin; ECG, epicatechin gallate; FA, ferulic acid; Isobutyrate, isobutyric acid; S, sucrose; LDP, agavins with a low degree of polymerization; MDP, agavins with a medium degree of polymerization; and HDP, agavins with a high degree of polymerization.

These phenolic markers were subsequently integrated with physicochemical, technofunctional, antioxidant, enzyme‐inhibitory, and SCFA variables in an unsupervised PCA (Figure 5) to support formulation selection under a global performance perspective. In this multivariate space, LDP5% was clearly positioned away from SCFA‐associated vectors (caproate and isobutyrate), indicating that its overall profile was not primarily driven by advanced carbohydrate oxidation or terminal fermentative metabolites. Instead, LDP5% clustered in a region characterized by the covariation of technofunctional structuring (apparent viscosity) and selected functional responses, including α‐amylase inhibition and redox‐related variables. Although LDP5% was not colinear with the apparent viscosity vector, the acute angle between these variables (< 90°) indicates positive covariation, supporting the interpretation of a more structured colloidal matrix. This behavior is mechanistically plausible, as body and viscosity in the kombucha analog are modulated by the combined contribution of bacterial cellulose (including dispersed fibrils or fragments) and soluble microbial exopolysaccharides. The yield and functional impact depend on substrate composition and fermentation conditions [38, 56, 57].

From a redox standpoint, the PCA further reflects the well‐documented assay‐dependent nature of antioxidant responses in fermented beverages reformulated with alternative carbohydrate sources. Rather than maximizing a single antioxidant endpoint, LDP5% exhibited a balanced multivariate signature in which DPPH contributed without dominating the projection, while ABTS showed more variable behavior. This pattern is consistent with previous studies reporting that changes in sugar source and matrix complexity redistribute antioxidant performance across assays rather than uniformly amplifying all responses [11, 14]. Accordingly, the antioxidant behavior of LDP5% appears compatible with selective remodeling of hydrolyzable tannins (e.g., ellagic acid rhamnoside and ellagic acid methyl ester) and fermentation cometabolites. This pattern supports its selection as a formulation in which technofunctional structuring and functional responses covary without dominance of terminal fermentation markers [58–60].

Crucially, this candidate profile was achieved without compromising microbial performance (Figure S1). Across 5% agavins formulations spanning LDP, MDP, and HDP, AAB, LAB, and yeasts displayed highly overlapping growth trajectories and reached final viable counts on the order of 10^7^–10^8^ CFU/mL. These levels are consistent with microbiologically stable kombucha fermentations [61–63].

**FIGURE 6:**
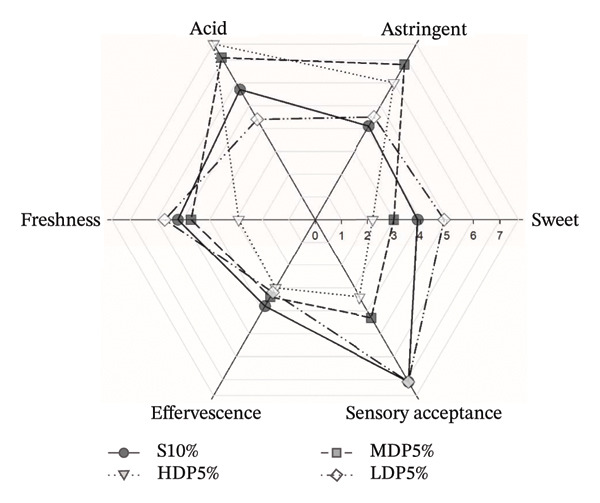
Radar plot of sensory attributes of kombucha analogs formulated with partial sucrose replacement by agavins of different degrees of polymerization. The sensory profile includes sweetness, acidity, astringency, freshness, effervescence, and overall sensory acceptance. Values correspond to mean scores obtained from a sensory panel (*n* = 10), allowing for a comparison of perceptual differences among formulations (S10%, HDP5%, MDP5%, and LDP5%). S, sucrose; LDP, agavins with a low degree of polymerization; MDP, agavins with a medium degree of polymerization; and HDP, agavins with a high degree of polymerization.

Finally, the sensory assessment confirmed that LDP5% attained the highest overall acceptability, aligning consumer preference with its balanced technofunctional structuring and integrated chemical/functional profile (Figure 6). This convergence supports LDP5% as the most robust prototype of the study under a chemistry–technology–microbiology–consumer framework, validating partial sucrose replacement with LDP agavins as a feasible route to develop lower‐calorie kombucha analogs with targeted functionality and high acceptability [61]. However, the relatively small number of biological replicates and the limited size of the trained sensory panel should be considered when interpreting these findings, as these factors may restrict the generalizability of the results.

## 4. Conclusions

This study demonstrates that partial sucrose replacement with agavins can enable the process‐based design of light kombucha analogs from mulberry coproducts while preserving technological functionality, microbial stability, and consumer acceptance under the conditions evaluated. The DP of agavins was found to modulate fermentation outcomes, with LDP agavins at 5% (LDP5%) promoting selective phenolic remodeling, balanced antioxidant responses, and favorable technofunctional structuring rather than extreme or stress‐driven profiles. Multivariate integration suggested that formulation performance depended on coordinated biochemical transformations rather than maximal responses in isolated variables. Importantly, agavins substitution did not compromise the viability or dynamics of the kombucha consortium, supporting fermentation controllability. This integrated chemical–technological–microbiological balance translated into superior sensory acceptance for LDP5%, indicating that LDP agavins may represent a promising strategy for developing reduced‐sugar fermented beverages with functional and consumer‐oriented value. Future studies should further explore the scalability of this formulation strategy and its effects on microbial dynamics and consumer acceptance in broader populations.

## Author Contributions

Conceptualization: Nuria Elizabeth Rocha‐Guzmán, Rosa Isela Ortiz‐Basurto, and Joshua David Castro‐Sánchez; methodology: Joshua David Castro‐Sánchez, Saúl Alberto Álvarez, Silvia Marina González‐Herrera, Karen Marlenne Herrera‐Rocha, and Blanca Morales‐Contreras; validation: Martha Rocío Moreno‐Jiménez; data curation: Nuria Elizabeth Rocha‐Guzmán, Joshua David Castro‐Sánchez, and Saúl Alberto Álvarez; funding acquisition: Nuria Elizabeth Rocha‐Guzmán; project administration: Martha Rocío Moreno‐Jiménez; writing–original draft preparation: Nuria Elizabeth Rocha‐Guzmán and Joshua David Castro‐Sánchez.

## Funding

This research was funded by the Tecnológico Nacional de México (TecNM) under Grant Number 22511.25‐P, by the Secretariat of Science and Humanities (SECIHITI), Mexico, through a postdoctoral fellowship (Grant Number BP‐PM‐20250505215034520‐10976755), and by a Master’s Degree Maintenance Scholarship (Grant Number CVU 2041201).

## Disclosure

All authors have read and agreed to the published version of the manuscript.

## Ethics Statement

The sensory evaluation protocol was reviewed and approved by the Research Ethics Committee of the Instituto Tecnológico de Durango (Approval Code CEI‐ITD/DP/2025‐001). Adult volunteers participated voluntarily after being informed of the study objectives and procedures, and informed consent was obtained. No personal or identifiable data were collected, participant confidentiality was ensured, and individuals were free to withdraw at any time. No vulnerable populations were involved in this study.

## Conflicts of Interest

The authors declare no conflicts of interest.

## Supporting Information

Additional supporting information can be found online in the Supporting Information section.

## Supporting information


**Supporting Information 1** Supporting Figure S1: Microbial growth kinetics during fermentation under different agavins degrees of polymerization (DPs), showing population dynamics of acetic acid bacteria (AAB), yeasts, and lactic acid bacteria (LAB).

**Supporting Information 2** Supporting Table S1: List of abbreviations and acronyms used throughout the manuscript.

## Data Availability

The data that support the findings of this study are available from the corresponding author upon reasonable request.
