# Supplementary material for: Process‐Based Design of Light Kombucha From Mulberry Coproducts: Effects of Agavins Degree of Polymerization on Physicochemical, Technofunctional, and Functional Potential
Source: Int J Food Sci. 2026 Apr 20;2026:2076539. doi: 10.1155/ijfo/2076539 (PMC13093542; doi:10.1155/ijfo/2076539)
Supplement: Supplementary file 1 — Supporting Information 1 Supporting Figure S1: Microbial growth kinetics during fermentation under different agavins degrees of polymerization (DPs), showing population dynamics of acetic acid bacteria (AAB), yeasts, and lactic acid bacteria (LAB). [file IJFO-2026-2076539-s002.docx]

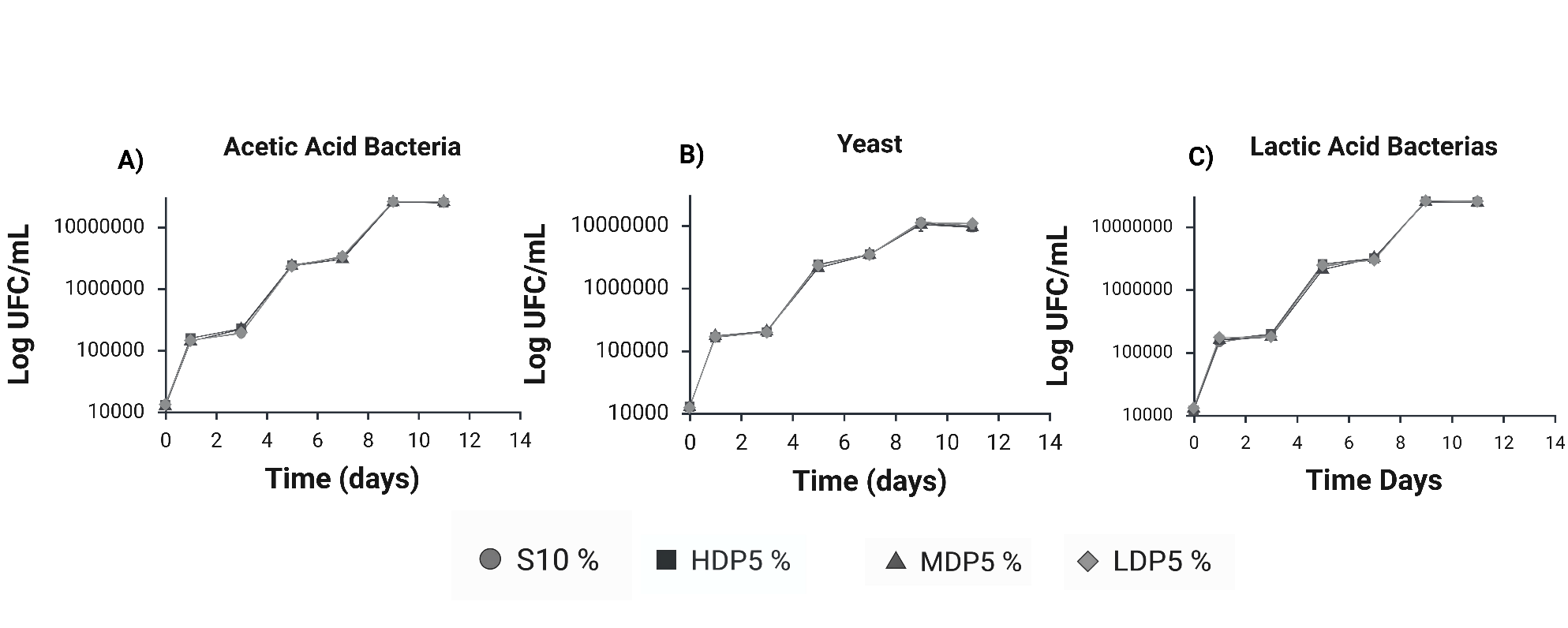


**Figure 1S.** Microbial growth kinetics during fermentation under different agavin polymerization degrees. Growth dynamics of (a) acetic acid bacteria (AAB), (**b**) yeasts, and (c) lactic acid bacteria (LAB) during fermentation under different agavin polymerization conditions: S10%, HDP5%, MDP5%, and LDP5%. Microbial populations are expressed as log CFU/mL as a function of fermentation time (days). For acetic acid bacteria, linear regression analysis showed significant growth trends for all formulations (p < 0.0001), with the following equations: S10% (y = 2,532,547.69x − 4,807,388.10; R² = 0.7376), HDP5% (y = 2,450,274.22x − 4,581,067.42; R² = 0.7333), MDP5% (y = 2,483,805.85x − 4,718,753.92; R² = 0.7352), and LDP5% (y = 2,531,570.54x − 4,788,477.05; R² = 0.7351). Similar growth patterns were observed for yeasts and lactic acid bacteria, characterized by a rapid increase in cell counts during the early fermentation stages, followed by stabilization at later time points. The consistency of growth kinetics across microbial groups indicates that fermentation time strongly influenced microbial proliferation, while variations in agavin polymerization degree modulated the magnitude of population growth.
