# Supplementary material for: Process‐Based Design of Light Kombucha From Mulberry Coproducts: Effects of Agavins Degree of Polymerization on Physicochemical, Technofunctional, and Functional Potential
Source: Int J Food Sci. 2026 Apr 20;2026:2076539. doi: 10.1155/ijfo/2076539 (PMC13093542; doi:10.1155/ijfo/2076539)
Supplement: Supplementary file 2 — Supporting Information 2 Supporting Table S1: List of abbreviations and acronyms used throughout the manuscript. [file IJFO-2026-2076539-s001.docx]

**Table S1.** Glossary

| µmol TE/mL | Micromoles of Trolox equivalents per milliliter |
| --- | --- |
| 3-NPH | 3-nitrophenylhydrazine |
| AAB | Acetic acid bacteria |
| ABTS | 2,2′-azinobis(3-ethylbenzothiazoline-6-sulfonic acid) |
| ANOVA | Analysis of Variance |
| App-Vis | Apparent viscosity |
| BEH | Bridged ethylene hybrid |
| C | Catechin |
| CaA | Caffeic acid |
| CA-glur | Cinnamic acid glucoronide |
| CouA | Coumaric acid |
| DNS | 3,5-dinitrosalicylic acid |
| DP | Degree of polymerization |
| DPPH | 2,2-diphenyl-1-picrylhydrazyl |
| EA | Ellagic acid |
| EAME | Ellagic acid methyl ester |
| EAR | Ellagic acid rhamnoside |
| ECG | Epicatechin gallate |
| EDC | 1-ethyl-3-(3-dimethylaminopropyl)carbodiimide |
| EPS | Exopolysaccharides |
| ESI | Electrospray Ionization |
| FCP | Free Choice Profiling |
| FRAP | Ferric reducing antioxidant power |
| HDP | High-DP |
| HM | High Mass |
| HSS | High Strength Silica |
| Iso-CouA | Isocoumaric acid |
| LAB | Lactic Acid Bacteria |
| LC-MS | Liquid chromatography coupled with mass spectrometry |
| LDP | Low-DP |
| LM | Low Mass |
| MDP | Medium-DP |
| MRM | Multiple reaction monitoring |
| MRS | De Man, Rogosa and Sharpe |
| MS/MS | Tandem Mass Spectrometry |
| ORAC | Oxygen radical absorbance capacity |
| Pa | Pascal |
| PA | Protocatechuic acid |
| Pa·s^n^ | Pascal second to the power n |
| PC | Principal Component |
| PCA | Principal Component Analysis |
| PERMANOVA | Permutational Multivariate Analysis of Variance |
| PLS-DA | Partial Least Squares–Discriminant Analysis |
| p-NPB | p-nitrophenyl butyrate |
| QA | Quinic acid |
| QDA | Quantitative Descriptive Analysis |
| Q-glu | Quercetin glucoside |
| Q-glur | Quercetin glucuronide |
| R | Rutin |
| S | Sucrose |
| SCFAs | Short-chain fatty acids |
| SCOBY | Symbiotic Culture of Bacteria and Yeast |
| SD | Standard Deviation |
| Sglur-Q | Quercetin sulfoglucuronide |
| ShA | Shikimic acid |
| SST | Stainless steel |
| t-CiA | Transcinnamic acid |
| TFF | Tangential flow filtration |
| THBA | Trihydroxybenzaldehyde |
| TQ-S | Triple Quadrupole – Sensitivity |
| Tris-HCl buffer | Tris(hydroxymethyl)aminomethane hydrochloride buffer |
| TSS | Total soluble sugar |
| UPLC | Ultra-performance liquid chromatography |
| VIP | Variable Importance in Projection |
| w/v | Weight per volume |
| YPD | yeast extract–peptone–dextrose |
